# Sharing sensitive data in life sciences: an overview of centralized and federated approaches

**DOI:** 10.1093/bib/bbae262

**Published:** 2024-06-05

**Authors:** Maria A Rujano, Jan-Willem Boiten, Christian Ohmann, Steve Canham, Sergio Contrino, Romain David, Jonathan Ewbank, Claudia Filippone, Claire Connellan, Ilse Custers, Rick van Nuland, Michaela Th Mayrhofer, Petr Holub, Eva García Álvarez, Emmanuel Bacry, Nigel Hughes, Mallory A Freeberg, Birgit Schaffhauser, Harald Wagener, Alex Sánchez-Pla, Guido Bertolini, Maria Panagiotopoulou

**Affiliations:** European Clinical Research Infrastructure Network (ECRIN), Boulevard Saint Jacques 30, 75014, Paris, France; Foundation Lygature, Jaarbeursplein 6, 3521 AL, Utrecht, The Netherlands; European Clinical Research Infrastructure Network (ECRIN), Boulevard Saint Jacques 30, 75014, Paris, France; European Clinical Research Infrastructure Network (ECRIN), Boulevard Saint Jacques 30, 75014, Paris, France; European Clinical Research Infrastructure Network (ECRIN), Boulevard Saint Jacques 30, 75014, Paris, France; European Research Infrastructure on Highly Pathogenic Agents (ERINHA AISBL), rue du Trône 98/Boîte 4B, 1050, Brussels, Belgium; European Research Infrastructure on Highly Pathogenic Agents (ERINHA AISBL), rue du Trône 98/Boîte 4B, 1050, Brussels, Belgium; European Research Infrastructure on Highly Pathogenic Agents (ERINHA AISBL), rue du Trône 98/Boîte 4B, 1050, Brussels, Belgium; European Research Infrastructure on Highly Pathogenic Agents (ERINHA AISBL), rue du Trône 98/Boîte 4B, 1050, Brussels, Belgium; Foundation Lygature, Jaarbeursplein 6, 3521 AL, Utrecht, The Netherlands; Foundation Lygature, Jaarbeursplein 6, 3521 AL, Utrecht, The Netherlands; Biobanking and Biomolecular Resources Research Infrastructure (BBMRI-ERIC), Neue Stiftingtalstrasse 2/B/6, 8010, Graz, Austria; Biobanking and Biomolecular Resources Research Infrastructure (BBMRI-ERIC), Neue Stiftingtalstrasse 2/B/6, 8010, Graz, Austria; Biobanking and Biomolecular Resources Research Infrastructure (BBMRI-ERIC), Neue Stiftingtalstrasse 2/B/6, 8010, Graz, Austria; Health Data Hub (HDH), rue Georges Pitard 9, 75015, Paris, France; Janssen Research and Development, Antwerpseweg 15, 2340, Beerse, Belgium; European Molecular Biology Laboratory (EMBL), European Bioinformatics Institute (EBI), Wellcome Genome Campus, CB10 1SD, Hinxton, Cambridgeshire, United Kingdom; Department of Clinical Neurosciences, Centre Hospitalier Universitaire Vaudois (CHUV), Rue du Bugnon 21, 1011, Lausanne, Switzerland; Center for Digital Health, BIH@Charité University Medicine, Anna-Louisa-Karsch-Straße 2, 10178, Berlin, Germany; Department of Genetics, Microbiology and Statistics, Universitat de Barcelona, Diagonal 643, 08028, Barcelona, Spain; Laboratory of Clinical Epidemiology, Istituto di Ricerche Farmacologiche Mario Negri IRCCS, Via GB Camozzi 3, 24020, Ranica (Bergamo), Italy; European Clinical Research Infrastructure Network (ECRIN), Boulevard Saint Jacques 30, 75014, Paris, France

**Keywords:** data sharing, FAIR principles, biomedical data, GDPR, centralized model, federated model

## Abstract

Biomedical data are generated and collected from various sources, including medical imaging, laboratory tests and genome sequencing. Sharing these data for research can help address unmet health needs, contribute to scientific breakthroughs, accelerate the development of more effective treatments and inform public health policy. Due to the potential sensitivity of such data, however, privacy concerns have led to policies that restrict data sharing. In addition, sharing sensitive data requires a secure and robust infrastructure with appropriate storage solutions. Here, we examine and compare the centralized and federated data sharing models through the prism of five large-scale and real-world use cases of strategic significance within the European data sharing landscape: the French Health Data Hub, the BBMRI-ERIC Colorectal Cancer Cohort, the federated European Genome-phenome Archive, the Observational Medical Outcomes Partnership/OHDSI network and the EBRAINS Medical Informatics Platform. Our analysis indicates that centralized models facilitate data linkage, harmonization and interoperability, while federated models facilitate scaling up and legal compliance, as the data typically reside on the data generator’s premises, allowing for better control of how data are shared. This comparative study thus offers guidance on the selection of the most appropriate sharing strategy for sensitive datasets and provides key insights for informed decision-making in data sharing efforts.

## Introduction

According to the FAIR guiding principles for scientific data management and stewardship, research data and associated metadata should be made ‘Findable’, ‘Accessible’, ‘Interoperable’ and ‘Reusable’ [1, 2]. Complying with these principles can be difficult when dealing with sensitive data, whether their sensitivity arises from their personal nature (e.g. healthcare data, biological samples and associated data, genetic data and clinical trial data) [3], intellectual property considerations (e.g. inventions, patents, software programmes, databases) [4, 5], requirements for access to Genetic Resources [6, 7] or the potential risk associated with making public certain data, such as those originating from Dual-Use Research of Concern (DURC) [8, 9].

When it comes to sharing and reusing sensitive data from living humans, there are major challenges in addressing ethical, legal and societal issues (ELSI). In the European Union (EU), the General Data Protection Regulation (GDPR, Regulation (EU) 2016/679 of the European Parliament and of the Council [3]) aims to protect EU citizens from privacy and data breaches in today’s data-driven society by setting out rules for the processing and movement of personal data. The GDPR recognizes the importance of large-scale data collection and analysis for scientific purposes stating in Recital 157 that *‘by coupling information from registries, researchers can obtain new knowledge of great value with regard to widespread medical conditions such as cardiovascular disease, cancer, and depression’*. The GDPR leaves room, however, for national derogations, and that has allowed some inconsistency in the legal landscape within the European Economic Area (EEA) to remain, adversely impacting the practice of clinical and scientific research [10–14]. The main uncertainties concern: (i) the concept of anonymization, specifically the circumstances under which pseudonymized data can be considered anonymized; (ii) the legal basis for processing personal data for secondary research and healthcare purposes; and (iii) the legal basis for cross-border (and especially outside the EEA) transfer of personal data for research and healthcare purposes. In addition, data governance is of major importance, as sharing and reusing sensitive data require clear policies and procedures, including clarity in data controllership and in the legal roles and responsibilities of all the entities involved [15–17].

Overcoming technical hurdles in the sharing and reusing of sensitive data poses significant challenges. For better findability and reusability, there is a need to establish shared vocabularies and multidisciplinary categorization systems suitable for describing the intricacies of sensitive data [18]. Furthermore, there is a requirement for technical infrastructures that provide robust and scalable data storage and access solutions, often including specialist software tools for management and analysis of sensitive data. Technical safeguards (e.g. anonymization [19] and encryption [20]) need to be implemented to ensure data security and data protection [21]. In addition, because sensitive data are often stored in different formats, reflecting different source systems, prior data harmonization may be required to support sharing and integration of those data [22, 23]. Although community-approved data standards exist that can help tackle the major issue of semantic and syntactic interoperability, in practice, the use of data standards remains low on a global scale and different standards are applied to different biomedical data types [e.g. Clinical Data Interchange Standards Consortium (CDISC) for clinical trial data, Observational Medical Outcomes Partnership (OMOP) for routinely collected healthcare data, Digital Imaging and Communications in Medicine (DICOM) for imaging data] [24]. Even when interoperability issues are overcome, data consistency and accuracy cannot be guaranteed. Thus, quality checks need to be in place to detect, for example, missing data points or errors [25, 26].

**Figure 1:**
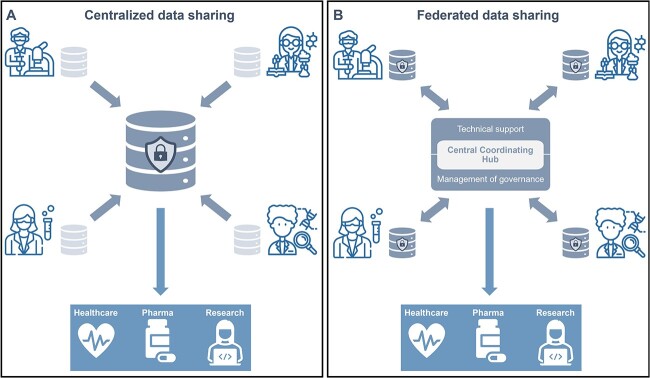
Sharing sensitive data in life sciences can follow centralized (**A**) or federated (**B**) approaches. Data generated by researchers in different domains, such as molecular biology, bioimaging, compound screening and genomics (represented clockwise from bottom left with icons created using Flaticon) can be deposited and accessed from a central repository, or in local ones for which access is often brokered via a central coordinating hub that federates data sharing.

Traditionally, research projects use a centralized data sharing model (Fig. 1A). Data are typically submitted to a single, central repository, where they are curated, managed and made available to authorized users. Large-scale examples in the life sciences include databases in genomics (e.g. the European Nucleotide Archive—ENA [27], ArrayExpress [28], the European Genome-phenome Archive—EGA [29]), epidemiology (e.g. the European Male Ageing Study—EMAS [30], the International Childhood Cancer Cohort Consortium—I4C [31]) and environmental research (e.g. European Soil Data Centre—ESDAC [32]) where substantial amounts of data from multiple sources need to be consolidated and made accessible to researchers. Additionally, numerous multi-centre research projects have set up joint data repositories typically managed by one of the consortium partners (e.g. in the VACCELERATE project clinical trial data management is handled by the Clinical Trials Data Centre in Cologne [33]). In many cases, using the centralized data sharing model may minimize data duplication and inconsistency, as well as improve data quality. It may, however, require users to overcome challenges such as disparate geographical locations of the data collection, the cost of the implementation and its sustainability beyond the project lifetime and compliance with a wide range of national (or even regional and institutional) rules and policies.

Technical improvements have made de-centralized or federated approaches (Fig. 1B) an attractive alternative to the centralized data model [34, 35], and a growing number of research collaborations in the life sciences are opting for federated models (e.g. the Federated EGA [36], the International HundredK+ Cohorts Consortium [37] and the Global Biodiversity Information Facility—GBIF [38]). With the federated approach, individual data sources maintain control over their own data, but agree to share some or all the information upon request. The goal of a federated data sharing model is to make infrastructure, data and in some cases policies available to all partners of a network, research initiative or distributed organization. Unfortunately, implementing a federated data sharing model often comes with challenges, such as the synchronization between data at source and those available for federated analysis, network connectivity between the data sources and the central coordinating hub, systems performance and maintenance and the identification of roles and responsibilities. In addition, a high level of transparency and scrutiny may be required to ensure that participating partners in the network have sufficient confidence in the security of the algorithms that access the data within their institutional firewall and in the responsible and lawful use of the data.

With the aim of providing guidance to life science researchers who wish to make sensitive data available for secondary use, we examine the specifics of centralized and federated data sharing models through the prism of five real-world, large-scale use cases: the French Health Data Hub (HDH), the BBMRI-ERIC Colorectal Cancer Cohort (CRC-Cohort), the Federated European Genome-phenome Archive, the OMOP/OHDSI network and the EBRAINS Medical Informatics Platform (MIP). The architectures of centralized and federated models for data sharing are compared, and the advantages and disadvantages of each approach are discussed. The selection of these particular use cases was influenced by their strategic importance within the European landscape and their pivotal roles in projects that shape European data sharing policies and practices. In particular, the HDH coordinates the HealthData@EU pilot project (https://ehds2pilot.eu/), the CRC-cohort is used as driving use case in the EOSC4Cancer project (https://eosc4cancer.eu/), the federated EGA provides requirements for Trusted Research Environments in the EOSC-ENTRUST project (https://eosc-entrust.eu/), OMOP is the framework promoted in the EHDEN project (https://www.ehden.eu/) and the MIP was a major outcome of the Human Brain Project (https://www.humanbrainproject.eu/en/).

### Centralized data sharing in the life sciences

#### The French HDH

**Figure 2:**
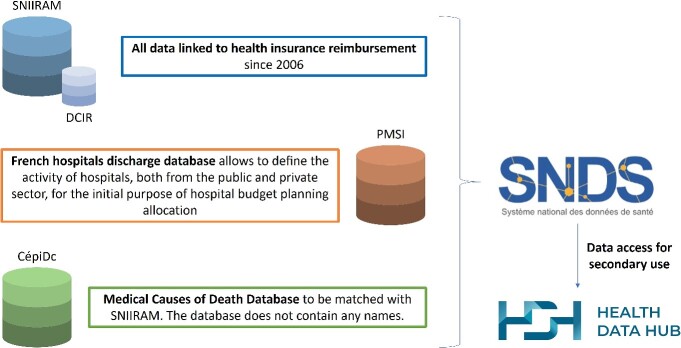
The 3 main components of the SNDS: SNIIRAM database (health insurance data), PMSI database (hospital data), Inserm’s CépiDC databases (medical causes of death).

The first example of centralized data sharing comes from the French HDH (https://www.health-data-hub.fr/). It was established as a public interest group [39] as part of the execution of a 2019 law on the organization and transformation of the healthcare system [40]. The group brings together 56 stakeholders, the vast majority from the public sector, to implement the strategic aims of the National Health Data System (SNDS, https://www.snds.gouv.fr/SNDS/Accueil). The SNDS links the main national health databases in France (Fig. 2) and contains:

(i) health insurance data (SNIIRAM database) and healthcare consumption and reimbursement data (DCIR database)
(ii) hospital data (PMSI database)
(iii) medical causes of death (Inserm’s CépiDC database)

The data in the HDH technological platform are pseudonymized, meaning that direct identifiers such as first name, surname, date and place of birth, address, etc., have been removed. Data in the HDH are not anonymized as this often involves data aggregation, which can be a barrier in answering certain medical research questions that require access to data with higher granularity. Since the data are not anonymous, important security measures need to be in place to guarantee that confidentiality is protected in compliance with the GDPR, the State’s Information Systems Security Policy and the SNDS security referential [41].

Organizational and technical security measures include encryption for data, storage space and workflows, with the HDH generating and managing its encryption keys using a proprietary hardware security module. Operating rights are segmented among HDH agents, called ‘operators’, each having pre-determined roles. Robust authentication mechanisms control access to the platform to prevent identity theft. Users access a secure workspace upon login, restricted to pre-validated project data with no administrative rights or internet access, allowing only anonymized results to be exported. The architectural design follows the ‘defence in depth’ principle, incorporating independent security components like flow filtering and malware detection. Trace analysis is performed, recording all actions by users and operators. Notably, health data resides in Microsoft data centres in the Netherlands that are compliant with EU regulations.

#### Data access

The data, within a well-defined research scope, can become accessible to projects contributing to the public interest, following a six-step approval process involving an independent committee called CESREES (Ethical and Scientific Committee for Research, Studies and Evaluations in the Health Sector) and the National Commission on Informatics and Liberty, known as the CNIL (https://www.cnil.fr/fr):

*Step 1*: Individuals who wish to access the data send a request to the HDH explaining how the data are necessary for a specific research project.

*Step 2*: The HDH sends the request to the CESREES, which is tasked to verify that the subject of the study is relevant and of public interest, that the data requested are relevant for the project and that the proposed methodology is scientifically robust.

*Step 3*: Based on these elements, which characterize the purpose of the data processing, the CNIL is requested to give its authorization, according to criteria of data protection and respect for citizens’ rights.

*Step 4*: Once the CNIL’s authorization has been obtained, the HDH consolidates the required data and prepares a secure ‘project space’ on its technological platform, which contains only the necessary data.

*Step 5*: Users of the technological platform have remote access to their ‘project space’ and process the data on the platform without being able to export it.

*Step 6*: The project results are made public on the HDH website, with due respect for academic and industrial competitiveness.

The ‘project space’ provided by the HDH is equipped with a variety of analytical tools such as JupyterLab, RStudio, Python, Cytomine, Superset and GitLab. To ensure semantic data interoperability, conversion to the OMOP Common Data Model (CDM) is performed. Projects deposited at the HDH focus on epidemiology and surveillance; disease pathways and care provision; medical, economic and social research; and research methodology.

#### The BBMRI-ERIC Colorectal Cancer Cohort

The Biobanking and BioMolecular resources Research Infrastructure—European Research Infrastructure Consortium (BBMRI-ERIC, https://www.bbmri-eric.eu/) was established in 2013 and is based in Graz, Austria. BBMRI-ERIC currently includes 24 countries and one international organization, the International Agency for Research on Cancer (IARC). BBMRI-ERIC aims to maximize the use of biosamples, images and health data for research into the prevention, diagnosis and treatment of disease. BBMRI-ERIC has a federated structure. The main entities involved are the biobanks, the national nodes (national contact point coordinating the activity in each country) and the BBMRI headquarters, with each entity having its own sovereignty. Similarly, the data architecture generally follows a federated model in which data stay at the source institutions, which legally remain the data controllers.

An exception to the federated data architecture of BBMRI-ERIC is the CRC-Cohort, a large-scale European initiative that aims to improve the understanding of colorectal cancer. The CRC-Cohort was developed within the EU-funded project ADOPT BBMRI-ERIC (https://www.bbmri-eric.eu/scientific-collaboration/adopt-bbmri-eric/) and, since the end of the project, has become a permanent asset of BBMRI-ERIC. The CRC-Cohort has gathered comprehensive datasets, together with tissue material from more than 10500 European colorectal cancer patients. Work started with the development of a common data model and a framework for data protection, the recruitment of data sources, the establishment of quality control and quality assurance procedures and finally the initiation of the data collection process, in 2017–18 [42]. More recently, a CRC-Cohort digital pathology imaging collection has been developed. It currently covers about 50% of the cohort and includes 26TB of Whole Slide Imaging (WSI) scans. To enable the cloud-readiness of the CRC-Cohort data and ensure interoperability with other data sources, work is now focused on a transition to the openEHR (https://www.openehr.org/) standard with HL7 FHIR and OMOP exports and for the WSI scans, a transition from proprietary formats to OME-TIFF.

#### Data access

**Figure 3:**
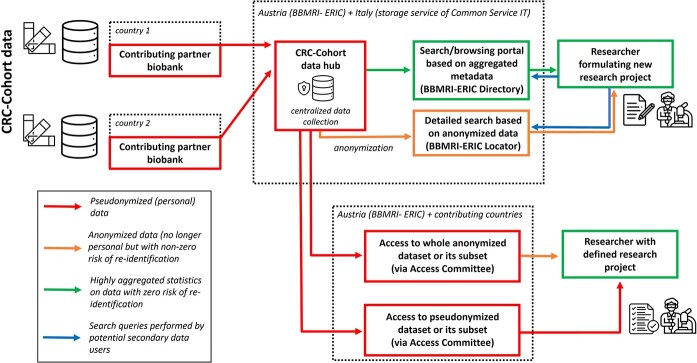
Overview of the CRC-Cohort data processing within BBMRI-ERIC and provisioning of access to data requesters (figure adapted from [42] using Flaticon).

BBMRI-ERIC acts as the CRC-Cohort data hub (Fig. 3). It becomes the data controller of the centralized data collection upon signature of a Data Transfer Agreement (DTA) with the contributing partner biobanks. Data access requests for secondary use of the CRC-Cohort data can be filed via the BBMRI Negotiator (https://negotiator.bbmri-eric.eu/) and are handled in accordance with the BBMRI-ERIC Access Policy [43]. To control access to the CRC-Cohort datasets, BBMRI-ERIC has set up an Access Committee comprising the data manager of the CRC-Cohort, one person appointed from BBMRI’s Common Service ELSI, to ensure ethical standards are met and one medical expert on colorectal cancer nominated by BBMRI-ERIC.

*Step 1*: The data manager checks whether the access request (or the project proposal) conforms to formal requirements: (i) the identity of the requester and their institutional affiliation are provided, and (ii) the project description is included in the request.

*Step 2*: If formal requirements are met, the medical expert assesses whether the request/project falls within the scope of the CRC-Cohort.

*Step 3*: If the request/project aligns with the cohort scope, then an ethics check is conducted.

*Step 4*: If the ethics check is successful, the Access Committee engages in consultation with relevant biobanks. Biobanks are given a 14-day window to veto the release of their data.

*Step 5*: If no vetoes are received within the specified period, the data release procedure is initiated. The data can be released under a DTA contract: (i) as a pseudonymized minimized dataset for a given purpose (default option), (ii) as a pseudonymized complete dataset and iii) as an anonymized dataset.

The CRC-Cohort is findable through the BBMRI-ERIC Directory (https://directory.bbmri-eric.eu/#/catalogue). For multi-criteria search of samples and datasets, the BBMRI-ERIC Locator (https://locator.bbmri-eric.eu/) can be used.

### Federated data sharing in the life sciences

#### The Federated European Genome-phenome Archive

The European Genome-phenome Archive is a public repository of human genomic and phenotypic data [29]. It was launched in 2008 as a service for the long-term and secure archiving and sharing of personally identifiable genetic, phenotypic and clinical data generated in the context of biomedical research projects or by research-focused healthcare systems [44]. The EGA is collaboratively maintained by the European Molecular Biology Laboratory’s European Bioinformatics Institute (EMBL-EBI) in the UK and the Centre for Genomic Regulation (CRG) in Spain, and since its inception, it has been operating under a centralized model. Data submitted to EGA are collected from individuals whose consent agreements authorize data release only for specific research use to *bona fide* researchers. Strict protocols govern how information is managed, stored and distributed by the EGA (https://ega-archive.org/about/ega/), and appropriate security measures are applied to control access and maintain individual confidentiality, while providing access to researchers and clinicians who have obtained authorization.

**Figure 4:**
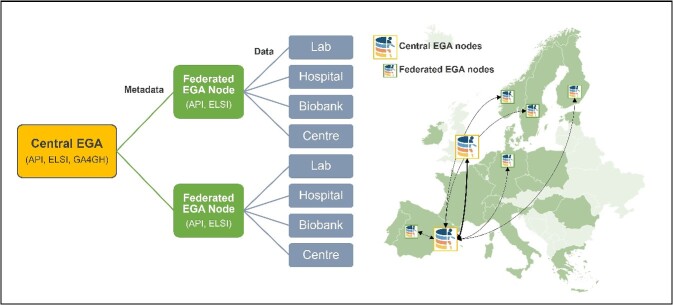
The Federated EGA Network links data resources. Each node in the Federated EGA Network stores and manages its own data locally and shares public metadata about the data it holds with the Central EGA, enabling researchers around the world to use a single-entry point to search for and discover data relevant to their research. API: Application Programming Interface; GA4GH: Global Alliance for Genomics and Health.

Recent advances in sequencing technologies and increased healthcare funding are now making it possible to use genomics in clinical practice. This shift means that much of the data are now coming from national or regional healthcare initiatives and in larger volumes. GDPR constraints, as well as other national legislations that restrict health data sharing outside local jurisdictions, led to the need for a non-centralized system to ensure data FAIRness, while respecting European and national data protection regulations. As a result, in 2016, discussions began to adapt the centralized EGA model to a federated model where EGA-like nodes could be deployed in other countries and be linked together in a network. In 2022, the Federated EGA consortium was officially launched after the first five national nodes signed Federated EGA Collaboration Agreements with the two Central EGA nodes [45] (Fig. 4). The first datasets hosted by the national nodes in Poland, Norway and Sweden have been made available in the Federated EGA in February 2024.

The vision for the Federated EGA is to be a network of connected data resources, typically nationally funded human data repositories hosted at research institutions or other research infrastructures, that provide transnational access to sensitive human data and thereby enables their secondary use. Each node in the Federated EGA Network stores and manages its own data locally and shares public metadata about the data it holds with the Central EGA. This model achieves multiple goals. First, the data remain within the jurisdiction where they were generated, thus facilitating adherence to the GDPR and any specific national legislation regulating cross-border data movement. Second, it removes the need to move data, potentially across long distances, a resource-intensive and potentially costly process. Finally, by centralizing the public metadata for all data within the Federated EGA Network at the Central EGA, researchers around the world can use a single-entry point to search for and discover data within the entire Federated EGA Network.

Data discovery can be done by simple text and keyword searches over the centralized public metadata; tools to support more complex querying are under development. One of these tools is the Beacon v2 [46, 47] standard for federated discovery of genomic and phenotypic data. Building upon its predecessor Beacon, Beacon v2 enables researchers to formulate complex queries such as ‘Do you have data covering allele X at genomic location Y from individuals diagnosed with disease Z?’. One option is for these queries to be distributed within the Federated EGA Network, and either yes/no or aggregated results can be returned. Thus, both the data and its discovery can be federated to support sensitive human data remaining in the jurisdiction under which it is regulated.

The federated EGA provides a range of services, including data submission, secure data storage and processing of data access applications. The nodes offer all EGA services (external helpdesk support, data submission services, computing and storage capacity, data distribution) for a particular jurisdiction and provide the same Application Programming Interfaces (APIs) and user interfaces as the Central EGA [36], enabling users to encrypt/decrypt datasets, upload/download, metadata submission, accession manager for secured and controlled access, format converter and quick browsing of EGA files. The nodes provide independent data distribution to users.

#### Data access

EGA (and federated EGA) uses a distributed data access management model. EGA datasets are affiliated to a Data Access Committee (DAC) that is responsible for all data access decisions. This means that decisions about access to studies are not made by the EGA, but by the DAC associated with each dataset. The DAC is generally composed of a committee formed by the organization that approved and monitored the original study, an institutional committee or an individual primary investigator [44]. The EGA contains over 6300 studies and works with over 1000 DACs.

Data access steps for datasets in the Federated EGA Network are still heterogeneous, reflecting different national requirements. However, in general terms, the main steps can be summarized as:

*Step 1*: Search for the study of interest in the EGA portal and identify the datasets that can answer a defined scientific question.

*Step 2*: Contact the corresponding DAC and request access.

*Step 3*: When access request is approved, detailed instructions are sent to requesters allowing them to download/access the requested datasets.

The requester may download the relevant dataset(s) to their computer via the EGA download client—pyEGA3. This process is contingent upon the applicant being issued with a personal EGA account associated with the relevant permissions. Furthermore, a data access agreement is made between the DAC and the applicant, which dictates how data can be used, stored or transferred once they are downloaded from the system. Files are encrypted, and users must decrypt them using Crypt4GH, a standard file container format from the GA4GH. This format allows genomic data to remain secure throughout their lifetime, from initial sequencing to sharing with researchers at external organizations.

#### The OHDSI/OMOP framework

OHDSI (https://www.ohdsi.org/) is a global interdisciplinary open science network whose goal is to improve human health and well-being by enabling the community to generate collaboratively evidence that supports better health decisions and better care [48]. The formation of the OHDSI community was motivated by the Observational Medical Outcomes Partnership (OMOP). This was a public–private partnership established in the United States to study the appropriate use of observational healthcare databases for assessing the effects of medical products [49]. The OMOP was chaired by the US Food and Drug Administration (FDA), administered by the Foundation for the National Institutes of Health (FNIH) and funded by a consortium of pharmaceutical companies.

Recognizing the technical challenges of conducting research using disparate observational databases, both in a centralized environment and in a federated research network, the team designed the OMOP CDM. It is a patient-centric model consisting of tables describing clinical data (e.g. drug exposure of a person), health system data (e.g. location of healthcare), health economics (e.g. cost), derived elements (e.g. chronological periods of condition occurrence), metadata (e.g. the CDM source used) and vocabularies (e.g. concepts), all in completely standardized terms. The OMOP CDM is designed to facilitate the analysis of observational healthcare data from diverse sources while preserving data provenance and is an extendable and evolving model.

OHDSI is an international initiative counting over 3000 collaborators in 80 countries (https://ohdsi.org/who-we-are/collaborators/) with a broad range of expertise, including biomedical informatics, epidemiology, statistics, informatics, health policy and clinical sciences, and records for approximately 928 million unique patients worldwide [50]. By leveraging large-scale observational data from Electronic Health Records (EHRs), insurance claims databases and other healthcare data sources, reliable scientific evidence can be generated on the natural history of disease, healthcare delivery and the effects of medical interventions [48]. The health data used in OHDSI research projects typically come from participating institutions that contribute their own de-identified patient data. Following a federated data model, controllership of the data is retained by the participating institutions.

Partners standardize their source data through an extract–transform–load (ETL) process into the OMOP CDM and apply OHDSI open-source tools securely behind their own firewall. OHDSI ensures homogeneous storage of observational healthcare data across different databases with interoperable formats and standard terminologies [51]. These are based on international classification of diseases codes, systematized nomenclature in medicine—clinical terms and normalized naming system for generic and branded drugs, for example.

In Europe, the EHDEN (standing for European Health Data and Evidence Network, https://www.ehden.eu/) project kicked off in November 2018 to support OHDSI’s objectives with the standardization and accessibility of observational health data across Europe. EHDEN focuses on transforming data from diverse EHR systems and other healthcare sources into the OMOP CDM.

In terms of user services, the OHDSI model focuses primarily on enabling collaboration and establishing an open community of harmonized data and standard analytics by developing open-source software, conducting methodological research and applying best practices across the OHDSI data network. OHDSI thus enables access to a range of resources, including standardized health records, analytics software, tools for evidence generation, data quality assessment, healthcare applications for various sectors and an open-source knowledge base for medical products.

OHDSI’s open-source data analytics and processing tools are numerous and include the OMOP-CDM, vocabulary mappings, a data quality dashboard, software for population health metrics, visualization platforms such as Atlas, pharmacovigilance platforms such as ADEpedia-on-OHDSI [52] and survival analysis methods. By harmonizing data into the OMOP CDM format, OHDSI enables large systematic studies, population-level estimates, drug and biomarker evaluations and patient-level predictions while maintaining data locality [51, 53], as protocols and analysis code can be executed across the network, with only aggregate summary statistics (not patient-level data) shared among collaborating researchers [48, 54].

#### Data access

**Figure 5:**
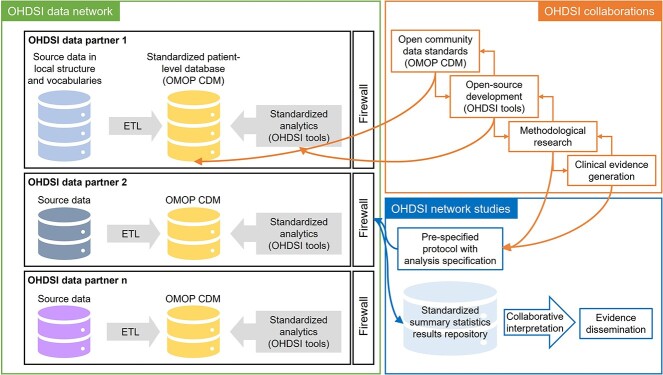
OHDSI community and research flow. OHDSI collaborators help establish open community data standards, develop open-source software, conduct methodological research and apply scientific best practices to both answer public health questions and generate reliable clinical evidence. OHDSI Network studies run across multiple CDMs at different institutions and offer the opportunity to investigate the effects of ‘real world’ factors on observational studies’ findings by examining a broad array of settings and data sources. ETL: extract, transfer, load. Adapted from [55].

Healthcare data in the OHDSI/OMOP network are not publicly available, and accessing the data requires following a specific set of procedures, as the data are housed in a secure network environment to protect patient privacy and ensure data security (Fig. 5).

*Step 1*: Obtain access to the OHDSI/OMOP network, which requires filling out the necessary forms and agreements.

*Step 2*: Once access is granted, the researcher identifies the dataset of interest and specifies which data are required.

*Step 3*: A data request is then submitted, which includes the research question or hypothesis, data requirements and any other relevant detail.

*Step 4*: The data request is reviewed by the OHDSI/OMOP team, and if approved, access to the data is granted.

*Step 5*: The researcher is then provided with instructions on how to access the data, which may include logging in to a secure portal, downloading the data or accessing through an API.

*Step 6*: Once accessed, data can be analysed using the tools and resources provided by the OHDSI/OMOP network.

Procedures for accessing the data may vary depending on the specific data source and project.

It is important to note that OHDSI focuses on providing tools, methodologies and standards for analysing observational health data rather than hosting or distributing datasets directly. However, OHDSI collaborates with various data partners and networks that contribute data for research purposes. These data partners may make their datasets available for analysis within the OHDSI framework, but access to specific datasets would depend on agreements and permissions established with the data owners. Researchers interested in accessing data through OHDSI need to join the OHDSI community and work within its collaborative framework. They may gain access to datasets through participating institutions, data partners or specific research projects within the OHDSI network.

#### The EBRAINS Medical Informatics Platform

The MIP is an open-source research platform developed to support the integration, analysis and sharing of health data in Europe via data federation [56]. It can be accessed through EBRAINS (https://www.ebrains.eu/), a sustainable Research Infrastructure (RI) dedicated to advancing brain research and neuroscience. The MIP enables clinicians, clinical researchers and clinical data scientists, as opposed to data scientists with programming intentions, to collaborate securely on sensitive human health data. Currently, it is installed in around 60 institutions across Europe.

The MIP Federations are organized according to specific diseases and are typically initiated by a consortium of researchers seeking to collaborate and share data without actual data transfer. Data harmonization and common data models facilitate federated analysis of large-scale, geographically distributed clinical datasets. At present, federations have been established for the domains of dementia, epilepsy, traumatic brain injury, mental health and stroke.

**Figure 6:**
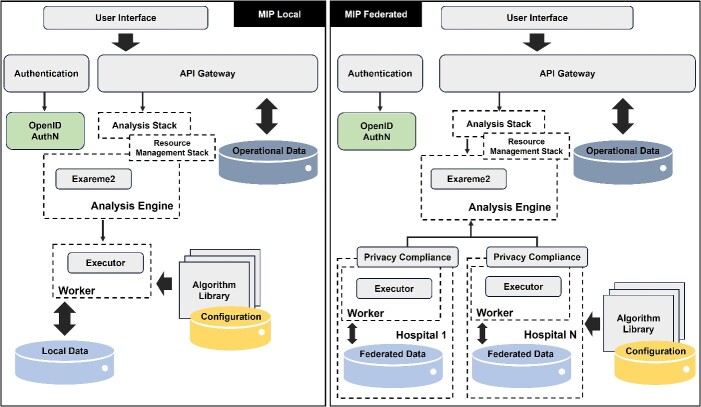
Local versus federated analysis at the MIP. Architecture and processing flow between the different modes of analysis offered. Workers: units that perform specific computations. Executors: components responsible for executing tasks locally in each node. Adapted from [60].

The MIP follows the principle of a two-tier architecture (Fig. 6), where the federated nodes (user side, holding the institution’s anonymized data) are representing the first tier. The central components of the federation containing, e.g. the user facing components, the central analysis engine, authentication/authorization components, represent the second tier, which executes the business logic for the web application. Each federated node is connected to the central node of the federation; any direct communication between the federated nodes is prohibited. Since release 7 of the platform, the infrastructure moved to a Kubernetes implementation, offering Secure Multi-Party Computation (SMPC) and Differential Privacy (DP) functionalities.

The MIP security system incorporates several components, including a secure Virtual Private Network (VPN) layer, data volume encryption, robust authentication (specifically, EBRAINS accreditation and Keycloak authentication, https://www.keycloak.org/), over-the-top encryption of data channels and outbound hospital connections. Importantly, no federated MIP is directly exposed to the public Internet. The central node functions as a firewall and proxy, ensuring data security, traceability and audit trails.

The ‘Local MIP’ offers an alternative deployment option, allowing an institution to install the MIP without federation connections. It mirrors the functionality of a federated MIP but lacks collaborative benefits with other institutions. Access is limited to locally accredited scientists.

As for user services, the MIP supports the integration and management of harmonized, structured tabular clinical data (e.g. lab results, EHR, phenotypic and demographic data) or features extracted from imaging (e.g. Magnetic Resonance Imaging—MRI). It provides a defined set of analysis and visualization tools, such as machine learning algorithms, statistical models and a visualization dashboard. Pre-built workflows and pipelines are available for common pre-processing and analysis tasks, and an API-gateway allows developers to build custom applications and integrations with other tools and services. Data remain in their original location; anonymized copies of datasets are uploaded to the MIP federated node, and queries can be run simultaneously on federated datasets across participating sites (principle of code visits data). Based on its built-in privacy thresholds, users retrieve aggregated query results.

#### Data access

Access to the MIP [57] is restricted to data users for activities within the scope of their approved use. Data users are also bound to comply with the EBRAINS access policy [58] when using the MIP.

Two levels of access exist for the MIP/EBRAINS Services and for which access protocols slightly differ: (A) Public Access or open access: the public MIP, installed on the EBRAINS RI, is available through EBRAINS accreditation and allows users to explore and analyse variables from synthetic datasets, mainly used for testing the platform functionalities. Recently, an anonymized real-world dementia dataset from the Leenaards Memory Centre (CLM) at the University Hospital of Lausanne was made available to the research community; data from a large Traumatic Brain Injury study (CREACTIVE [59]) are in the process of integration. (B) Restricted/controlled access: access to the relevant federations is granted on a case-by-case basis and requires a double authentication protocol.

*Step 1*: When registering to use the MIP for the first time, users will be prompted to create an EBRAINS account. These credentials are needed to complete the registration on the MIP.

*Step 2*: Users must complete the registration by supplying the EBRAINS account, name, institutional e-mail address and motivation for using the MIP.

*Step 3*: Following evaluation, the committee representing the contributing institutions within a federation will grant access to the data. Users can request to set up new federations in areas of their scientific interest. Upon establishing or joining a federation, the data provider’s institution signs agreements with Centre Hospitalier Universitaire Vaudois (CHUV). Upon MIP Network admission, accredited researchers, can query MIP federated Nodes to obtain aggregated results.

Of note, the MIP provides access to data through federated analysis, which means that researchers can explore and analyse distributed data (from multiple, often geographically separated datasets) within the MIP secure environment without the need to transfer the data.

## Discussion

Here, we present a comprehensive overview of the two main models of sensitive data sharing: centralized and federated, illustrated through five real-world use cases. However, these examples represent only a few of the many initiatives underway. We also outline the strengths and limitations of each approach and compare them against a number of key criteria, aiming to guide data holders in selecting the optimal sharing strategy for their datasets and at providing invaluable insights for informed decision-making in data sharing endeavours. Given the scarcity of information and comparative studies on strategies for sharing sensitive data, our analysis not only addresses a critical knowledge gap in the domain but also serves as a vital resource for stakeholders across the data lifecycle.

Before the launch of the Human Genome Project (HGP) in the 1990s, ‘there had been no serious discussion of data sharing in biomedical research’ [61–63]. But at a 1996 summit in Bermuda, leaders in the scientific community agreed on what became known as the ‘Bermuda Principles’, which ensured that countries or scientists did not withhold data that were important for the success of the HGP. These principles called for all DNA sequence data to be published in public databases within 24 hours, without exception, and were revolutionary in that, until then, the norm had been for researchers to keep their data as long as they could and only make them available after publication. Later, in 2003, scientists and professionals in biomedicine issued a public declaration known as the Fort Lauderdale Agreement supporting the free and unrestricted use of genome sequencing data by the scientific community before that data are used for publication and aiming for a shared system of responsibility among funding agencies, resource producers and resource users to maintain and expand a community resource of genomic data [61]. Together with the Bermuda Principles [64, 65], the agreement represented a significant step forward that marked the beginning of wider data sharing in biomedical research and the founding documents of open access models in the field.

Over 20 years later, the concept of data sharing and open access has been extended beyond HGP to other projects and types of sensitive data, including health data, and today, the management, stewardship, sharing and re-use of data are increasingly adhering to the FAIR Guiding Principles [1]. Applying the FAIR principles in the field of health contributes to improved patient outcomes, delivery of medical care and scientific discovery and innovation. The FAIR principles ensure that health data are findable by both humans and machines, facilitating efficient data retrieval and enabling researchers, clinicians and policymakers to locate relevant datasets quickly, thereby enhancing the speed and effectiveness of research, clinical decision-making and public health initiatives. The adoption of standardized formats, terminologies and protocols encourages data harmonization and integration. Interoperable health data facilitates data sharing, aggregation and analysis, supporting interdisciplinary research, population health management and healthcare innovation. Finally, the FAIR principles emphasize the importance of ensuring that data are reusable for future purposes. By addressing issues such as data quality, documentation and licensing, FAIR-compliant health data will remain valuable assets over time. Wide adoption of FAIR practices is expected to improve the so-called ‘reproducibility crisis’ in the biological and medical field [66].

Despite progress, concerns about protection of privacy and confidentiality of the data subjects often lead to policy responses that restrict access to data. The restrictions are much more complex when it comes to large-scale multinational research collaborations. As a matter of fact, researchers need to take into consideration different regulations, policies and recommendations, including the GDPR that superseded the Data Protection Directive 95/46/EC [67] in Europe, the Artificial Intelligence Act (AI Act) [68], the Health Insurance Portability and Accountability Act (HIPAA) [69] in the United States, the World Medical Association’s Declaration of Helsinki on ‘Ethical Principles for Medical Research Involving Human Subjects’ [70] and the Council of Europe ‘Convention on Human Rights and Biomedicine’ (Oviedo Convention) [71] to name but a few. In addition, and despite harmonization efforts such as the EHDEN project [72] or GA4GH [73, 74], national data hubs, healthcare centres and research institutions often use customized system architectures, data formats, data standards and cybersecurity protocols, which makes technical interoperability between data infrastructures in Europe and beyond challenging [75].

Harnessing the wealth of information contained in existing datasets is thus increasingly essential in biomedical research, innovation and policy. Therefore, data need to be accessible for re-use and sensitive data sharing strategies need to be carefully considered. Moreover, increasing pressure for openness and adherence to the FAIR principles, highlights the importance of selecting data sharing approaches that are suitable for the respective datasets, as, for example, not all health data are suitable for unrestricted access due to legal constraints and privacy considerations.

As a generalization, the centralized model presents a multitude of advantages for sharing sensitive data, making it an attractive choice for researchers and organizations. First and foremost, it is the data sharing model that most researchers are familiar with, ensuring a quicker adoption from the different scientific communities and a more effective data re-use. Additionally, centralized models simplify retrospective data integration through conversion to interoperability standards or data harmonization (e.g. the French HDH converts to OMOP CDM), facilitating collaboration among diverse research teams. Improved data findability is another advantage, as centralized data repositories are typically easily searchable, accompanied by detailed (meta-)data catalogues (e.g. the CRC-Cohort information is available in the BBMRI Directory and BBMRI Locator). Furthermore, centralization facilitates data quality control and, as a result, enhances data quality, ensuring that data re-users are working with reliable information. The centralized approach also enables a faster and simpler data access process with established access policies, reducing bureaucratic obstacles.

Despite their advantages, there are concerns about the sustainability of centralized models, primarily due to the challenges associated with handling sensitive data. These challenges can render centralization impractical and often unfeasible, particularly on a multinational scale. The sensitive nature of the data often necessitates explicit consent for sharing across institutions and borders due to various national, regional and institutional rules and regulations safeguarding personal data and health information. Efforts to address these concerns through anonymization protocols can often result in data becoming unusable for research, owing to added noise and information loss. Additionally, the technical demands and costs associated with transferring and storing data in a single location pose significant limitations, given the enormous volume of health data generated by research institutes and healthcare facilities today. Furthermore, when centralized repositories are created in the frame of a research project collaboration, a copy of the data of each contributing centre needs to be transferred and stored within the repository, raising concerns about the longer-term financial sustainability of these services once the project funding is over.

To cater to the limitations of the centralized data sharing model, federated architectures for sensitive data sharing have started to emerge over the last decades. In the federated data sharing model, ELSI compliance and consent management are simplified as the data remain at the source centres, making it a suitable solution for sharing and re-use of highly sensitive data like EHR, while ensuring that data providers maintain local control over the data. Federated analysis is well suited to the re-use of data, but not to the primary use of a database, as it does not offer the degree of freedom needed to exploit the data, create new variables and combine new scores and indicators. Thus, for primary use of the data, e.g. in clinical trials and cohort analyses, a centralized model is better suited and preferred in practice by most researchers. However, the federated model is nowadays a workable solution for the analysis or mapping of different existing cohorts or disease registries (e.g. the federation of European registries for stroke in the MIP). Finally, when several types of data are involved (sequencing, imaging, etc.), as is the case for standard biological data, a federated model is much more appropriate than a centralized one, which is more commonly applied to hosting of a single data type. Scalability of federated systems can rather easily be achieved through inclusion of additional nodes (e.g. as currently explored within the Federated EGA Network and in MIP through EBRAINS partnerships), and as such, it is the preferred choice for managing vast volumes of data. Additionally, keeping the data up to date is more manageable in a federated model.

However, the federated data sharing model is not without its challenges. For retrospective data collections, it presents limitations in data interoperability and harmonization across participating nodes, which can hinder efforts to integrate data effectively. Inconsistent data quality policies across nodes can further complicate data management. Identity management across various nodes can become more complex (e.g. in the case where users need to authenticate separately with each participating system to access distributed data), potentially leading to extended wait times. Additionally, incentives are needed to encourage the recruitment of new participating nodes, especially since there are often concerns about security issues due to the risk of running malicious algorithms behind institutional firewalls. Nevertheless, it is possible in a federated system to reasonably streamline data access and management as in a centralized system, even if this requires additional resources and coordination. If within a federated system there is a centralized core of processes, interfaces and data standards (e.g. as done within the OHDSI network), these can be systematically implemented in the participating federated nodes. Table 1 summarizes advantages and disadvantages of centralized and federated models for sharing sensitive data in life sciences.

**Table 1:** Advantages and disadvantages of the centralized and federated models

	Centralized	Federated
Advantages	+ The model that most researchers are familiar with + Easier data linking/harmonization/interoperability (especially for retrospective collections) + Improved data findability + Easier data quality control & improved data quality + Faster/simpler data access process & standardized data access policy + Easier to incentivize data donation/provision + Easier data analysis (e.g. no need to install software locally per project) + Easier cross-charging of compute costs to end users	+ Easier ELSI compliance & consent management + Allows sharing and reuse of highly sensitive data (e.g. medical records) + Data providers retain local control over data + Model more easily scalable + Best suited model for big data volume + More robust long-term sustainability of the data source depending on institutional funding + Easier to keep the data up to date + Can mitigate challenges related to varied legal frameworks and data protection regulations across countries
Disadvantages	− Big data volume challenging to handle & trained personnel required − High cost for developing a secure centralized infrastructure − ELSI compliance & consent management challenging (e.g. due to different national rules) − Data security concerns/important consequences in the event of data breach due to the amount of data contained in a single repository − Challenging to gain trust over data governance − Data synchronization: data only as recent as the latest upload − Hard to sustain long term; often dependent on project funding	− Limited data interoperability/data harmonization more challenging (especially for retrospective collections) − Heterogeneous data quality policies across participating nodes − More challenging identity management across nodes − Multiple data access policies may apply/extended times for data access − Need for investment/coordination of an intermediate interface across participating nodes − Incentives needed for the recruitment of new participating nodes − Concerns for security issues or malicious algorithms running behind firewall (in cases where this is permitted) − Complex cross-charging to users of compute resources used

While Table 1 provides a comprehensive listing of the advantages and disadvantages associated with each approach, the reality is often more nuanced and different use cases can have different specificities. In practice, hybrid models are frequently implemented, leveraging the strengths of both centralized and federated models. As an example, the Swedish Cohort Consortium (Cohorts.se) [76] centralizes the data of participating cohorts at the Uppsala Clinical Research Centre. Participating cohorts follow common harmonization guidelines according to Maelstrom Research and the data access requests are managed centrally but distributed to the participating cohorts as relevant. When it comes to research collaborations requiring the analysis of harmonized data, three approaches are implemented: pooled data analysis, summary data meta-analysis and federated data analysis. The first two approaches, pooling individual-level data in a central location and meta-analysing summary data, are commonly used in multi-centre research projects. To address data privacy and confidentiality concerns and enable international collaborations, the consortium has more recently applied DataSHIELD for the federated analysis of geographically dispersed datasets. DataSHIELD [77] enables data analysis without exposing the raw data to re-users, a strategy also implemented in the MIP through its analysis engine, Exareme 2 [78].

To help researchers determine which data sharing approach is best suited to their scientific question and related data types, Table 2 provides a comparison of the two models based on several criteria such as data location, data governance, data control, data access, data protection, data security, data interoperability, data processing and analysis, data quality, data usability, scalability and sustainability.

**Table 2:** Comparison between the centralized and the federated models

Criteria	Centralized	Federated
Data location	▪ Sensitive data are stored and managed in a central repository.	▪ Sensitive data are distributed across multiple decentralized locations or institutions.
Data governance	▪ Effective frameworks are necessary to establish rules, policies and protocols for data access, sharing, quality assurance and compliance within the centralized system.	▪ Data governance is often indistinct. Best-practice examples develop policies, protocols and agreements that define how data are shared, accessed, used and protected among the participating institutions.
Data control	▪ In most cases, source centres retain control of their data but share stewardship with the centralized data repository. In some cases, joint controllership is established between the source centres and the centralized data repository.	▪ Source centres retain control and stewardship of the data.
Data access	▪ Access to the data is typically granted by the centralized repository to authorized individuals or organizations through predetermined processes, permissions and agreements.	▪ Access to data is controlled by each participating institution, allowing them to determine which individuals or organizations can access their data and for what purposes.
Data protection	▪ The centralized system is fully responsible for privacy and security of the data. ▪ Data protection and security measures must be robust to protect the centralized repository from unauthorized access or breaches. ▪ Higher impact in the event of a breach, as all the data are stored in one location.	▪ Each node is responsible for the security of its own data. ▪ Federated models enhance data protection by keeping sensitive data within the source centre. Often, access is limited to only aggregated data or summary statistics. ▪ Reduced amount of data that could be accessed through a breach, due to the diffuse nature of the data.
Data security	▪ Strong security measures, including encryption, access controls and authentication mechanisms, are necessary to protect the centralized repository from unauthorized access and data breaches.	▪ Local systems must have robust security measures in place to protect sensitive data, including encryption, access controls and secure data transfer protocols, to ensure privacy and prevent unauthorized access.
Data interoperability	▪ Requires mechanisms to integrate data from different sources and formats, ensuring interoperability and consistency.	▪ Requires mechanisms to harmonize data across different institutions, addressing variations in data formats, coding systems and terminologies to facilitate meaningful analysis.
Data processing and analysis	▪ Data analysis and processing are performed within the central repository using its computational resources.	▪ Data analysis is performed locally at each institution using their own computational resources, and only aggregated or summarized results are shared.
Data quality	▪ High potential for data standardization and integration, which leads to consistent and uniform data. ▪ Quality control measures, such as data validation checks, data profiling and data cleansing techniques, are necessary to identify and rectify data quality issues within the central repository.	▪ Ensuring data consistency and accuracy across institutions is challenging and requires more efforts to harmonize data across different institutions. ▪ Each participating institution is responsible for ensuring the quality of its own data. Institutions may implement local data quality control processes, including data validation, data cleansing and error correction, to improve data quality within their own systems.
Data usability	▪ Data stored in one location, facilitates access, retrieval, streamlined analyses and integration. ▪ Checking the quality of source data is simple.	▪ Accessing and integrating data across institutions requires additional coordination and data harmonization efforts. ▪ Inspecting the source data if there is any doubt about the validity of the analysis is more difficult.
Scalability	▪ Scalability is challenging as the volume of data increases, requiring additional infrastructure and resources. ▪ Often, an additional data copy is needed (one at the source centre, one at the centralized repository).	▪ Inherently scalable as the computational workload is distributed across participating institutions, making it easier to handle large volumes of data. ▪ No need for an additional data copy.
Sustainability	Relies on: The availability of adequate resources and ongoing maintenance of the infrastructure. Sufficient funding and budget allocation to cover the ongoing expenses. Expanding the infrastructure to accommodate increasing data volumes and processing. Cross-charging compute costs based on straightforward 1:1 agreements between repository and user.	Relies on: ▪ Costs for maintaining data storage are usually already covered by the ongoing obligation to preserve a copy of the dataset. ▪ Continued engagement, trust-building and shared goals are vital for maintaining the federated system over time. ▪ Fair resource distribution, including the availability of adequate computational power, storage and expertise. ▪ Cross-charging compute costs based on agreements with all nodes in the federated network.

It is important to note that the European sensitive data sharing landscape is rapidly evolving and emerging regulations, infrastructures and policies are likely to significantly affect decision-making processes regarding data sharing model selection in the near future. In this regard, one major initiative is the European Open Science Cloud (EOSC) [79], which is creating since 2015 a federated virtual environment for global access to research data and tools, fostering collaboration across borders and disciplines and aiming to advance research, innovation and education in the European Research Area. For the period 2024–27, research will focus on Trusted Research Environments (TREs) for sensitive data with the application of federated approaches. Funded projects under HORIZON-INFRA-2023-EOSC-01-06 ‘Trusted environments for sensitive data management in EOSC’ [80] are tasked to address some of the inherent challenges of the federated model, such as improving data interoperability across different European TREs, harmonizing data quality and data access policies and simplifying identify management across different systems. Recognized European TREs, such as the Norwegian Sigma2 (https://www.sigma2.no/) and the Safe Haven of the University of Dundee (https://www.dundee.ac.uk/hic/safe-haven), are engaged with the aim of harmonizing and improving current practices.

Over the last couple of years, Europe has witnessed a policy priority shift towards a digital era. This was marked by the ‘Path to the Digital Decade’ [81], initially proposed by the Commission in 2021, which has now evolved into the ‘Digital Decade Policy Programme 2030’ that came into force in January 2023 [82]. Of relevance to biomedical data is the decision of the European Commission to prioritize the creation of a European Health Data Space (EHDS), introduced in May 2022 through a legislative proposal [83]. EHDS foresees a hybrid approach based on health data centralization at the national level and federated access to that data for research purposes via the use of Secure Processing Environments (SPEs). The development of a common infrastructure, HealthData@EU, is planned to support the secondary use of health data and offering alignment in terms of data governance, data management, data interoperability, data findability and data quality.

In conclusion, the evolution of data sharing in life sciences, particularly in the context of genomics and routinely collected health data, has undergone transformative changes in recent decades. Driven by pioneering initiatives such as the Bermuda Principles and the FAIR Principles, the field has moved from a culture of data hoarding to one of open science, paving the way for broader data sharing practices. The subsequent emergence of centralized models has provided effective means of collaboration, data integration and quality control, but has raised issues of long-term sustainability and of handling cross-border sharing of highly sensitive information. The introduction of federated architectures has addressed some of the limitations by allowing local control and scalability but has raised issues of retrospective interoperability and incentivizing participation. Hybrid models, meanwhile, illustrate the practical integration of centralized and federated approaches. As the European landscape continues to evolve, the EOSC and the EHDS are poised to shape the future of sensitive data sharing, underlining the importance of federated and centralized elements for effective, secure and sustainable research efforts. With the guidance provided in this manuscript, researchers are now faced with a nuanced decision-making process, based on factors such as data governance, security and ease of use, as illustrated by the detailed criteria provided in Table 2. The ongoing pursuit of innovative solutions, including federated analysis tools and AI, reflects a dynamic effort to balance the benefits and challenges inherent in the evolving landscape of sensitive data sharing in the life sciences.

**Key Points**

- The manuscript assesses and compares the characteristics of centralized and federated data sharing models across five use cases: the French Health Data Hub, the BBMRI-ERIC Colorectal Cancer Cohort, the federated European Genome-phenome Archive, the OMOP/OHDSI network and the EBRAINS Medical Informatics Platform.
- Most researchers working in life sciences are accustomed to the centralized data sharing model. Typically, centralized models facilitate data linking, harmonization and interoperability of the data, especially for asynchronous data collections. Data quality processes are more easily applied, and there is a standardized data access policy, resulting in most cases in faster data access. Despite these advantages, ELSI compliance can become cumbersome in multinational collaborations due to coping with different legislations in the countries of data collection. Also, data security is of highest importance due to the consequences in the event of data breach given the high amount of data contained in a single repository.
- Federated data sharing models provide easier ELSI compliance as the data often reside at the data controller’s premises, allowing them to have control and oversight of how and with whom their data are shared. Federated models have recently allowed secure data sharing of highly sensitive data types, such as Electronic Health Records. Federated architectures are easily scalable and suitable for big data volumes. For asynchronous data collections, and in case common data models have not been applied at the beginning of the study, data interoperability and harmonization are more challenging. For federated systems, it is recommended that a central coordinating hub is created, harmonizing data access and quality policies.
- Future directions in the European sensitive data sharing landscape are shaped by emerging regulations, infrastructures and policies. With the advent of the European Open Science Cloud, anticipated developments in Trusted Research Environments are poised to enhance data management practices and foster cross-border collaboration. Moreover, the European Digital Decade Policy Programme 2030 and the establishment of the European Health Data Space (EHDS) underscore Europe’s commitment to advancing digitalization in healthcare and research. Hybrid models for sensitive data management are likely to play an important role in accelerating such digitalization. These models offer a more modular approach that combines advantages from both the centralized and federated models, which is likely to solve many of the inherent drawbacks of current systems.

## Data Availability

No new datasets were generated in this work.

## Author Biographies

**Maria A. Rujano**, PhD in Medical Sciences from the University of Groningen, serves as a Project Manager at ECRIN contributing to EU initiatives for healthcare data research. With expertise in cell and neurodevelopmental biology, she has worked at prestigious research centres, including Institute Curie and Institute Pasteur.

**Jan-Willem Boiten**, PhD in Chemistry, is a Senior Program Manager at Lygature. With over 20 years of experience in multicentre collaborations across life sciences, he is coordinating large-scale biomedical data infrastructure programs in the Netherlands as well as at the European level.

**Christian Ohmann** has a PhD in Mathematics, an interim examination in medicine and a habilitation in the field of ‘Theoretical Surgery’. He was the head of the Coordination Centre for Clinical Trials (KKS) at the Medical Faculty of the Heinrich Heine University Duesseldorf, Germany (1999–2014) and currently consults ECRIN on clinical trial data sharing and chairs ECRIN’s Network Committee.

**Steve Canham**, a senior data manager at ECRIN, brings 18 years of expertise in clinical trial data systems and management. Formerly, he led the IT team at the Institute of Cancer Research, London (2002–11).

**Sergio Contrino** is the Head of Data Projects at ECRIN. An Electronics Engineer by training, he has previously worked as a senior software developer at the University of Cambridge, Genetics department, and at the European Bioinformatics Institute (EBI).

**Romain David**, PhD in Oceanography and Data Mining, serves as a Research Fellow and Data Steward at ERINHA. With 20 years of expertise in environmental information systems, he specializes in data mining, graph approaches and FAIR-compliant data management. He contributes as a co-chair to Research Data Alliance (RDA) Interest Groups (e.g. sensitive data).

**Jonathan Ewbank** joined ERINHA as Director General in 2021, after 25 years as a group leader at Centre of Immunology of Marseille-Luminy (France).

**Claudia Filippone** is a virologist working as a senior Scientific Programme Manager at ERINHA. Her research experience focuses on viral emergence and zoonoses, both in Europe and Africa.

**Claire Connellan**, a European Project Manager at ERINHA, focuses on the Integrated Services of Infectious Disease Outbreak Research (ISIDORe) project, that aims to enhance pandemic preparedness. With expertise in project management and policy development, she holds an MA in International Peace and Security from King’s College London.

**Ilse Custers** is a pharmacochemist (VU Amsterdam) and health economist (Pompeu Fabra University) by training. She is currently working as a Program Manager in Lygature managing projects that build infrastructure for health and genomic data sharing.

**Rick van Nuland**, PhD, is a Program Manager in Lygature focusing on health research infrastructures and trained as a Molecular Cancer Biologist at University Medical Center Utrecht and Stanford University.

**Michaela Th. Mayrhofer** is a social scientist by training. Her PhD studied the modes of governance of biobanks. Her research focus today lies on GDPR compliance, ethics of AI and establishing and promoting ELSI best practices in biomedical research. Since 2019, she is leading BBMRI-ERIC’s ELSI Services & Research unit.

**Petr Holub** is an Associate Professor of computer science at Masaryk University. Since 2015, he has become Chief IT Officer at BBMRI-ERIC. He has background both in sciences (MSc with specialization on computational quantum chemistry in 2001) and computer science (PhD in 2005, specializing on computer networks, self-organizing systems and multimedia processing).

**Eva García Álvarez** works as an IT Scientist and Project Manager at the BBMRI-ERIC. She obtained her PhD in Molecular Medicine from Universidade de Santiago de Compostela where she investigated the genomic landscape of somatic viral integration in cancer.

**Emmanuel Bacry** is a graduate of École Normale Supérieure (ENS) and holds a doctorate in Mathematics. He is currently CNRS Research Director at Paris-Dauphine University and Scientific Director of the French Health Data Hub. He led the Big Data & Data Science Initiative at Ecole Polytechnique for more than 4 years (2016–19).

**Nigel Hughes**, Scientific Director at Janssen, boasts a 35-year career across the NHS, NGOs, patient organizations and the pharmaceutical industry. His diverse experience encompasses clinical work in HIV and viral hepatitis, various roles in pharmaceuticals and leadership in health data initiatives like EHDEN and EMIF.

**Mallory A. Freeberg**, Coordinator for the European Genome-phenome Archive (EGA), holds a PhD in Bioinformatics from the University of Michigan. Her research focused on post-transcriptional gene regulation in model organisms like *Caenorhabditis elegans* and *Saccharomyces cerevisiae*. She further explored protein:RNA interactions during her postdoctoral fellowship at Johns Hopkins University.

**Birgit Schaffhauser** works as a Senior Project Manager at CHUV, overseeing the coordination of Medical Data Analytics Services using the Medical Informatics Platform (MIP) and the Human Intracerebral EEG Platform (HIP). She holds a PhD in Biology from Vienna University, and her research focused on transgenic expression of the angiogenesis inhibitor endostatin during β cell tumorigenesis.

**Harald Wagener** has worked in the IT industry for 20 years, building and operating large scale and distributed cloud infrastructures. He is currently the Head of Cloud and IT at the Berlin Institute of Health (BIH) at Charité.

**Alex Sánchez-Pla** is Full Professor in the Department of Genetics, Microbiology and Statistics at the University of Barcelona. He is also the former Head of the Statistics and Bioinformatics Unit (UEB) of the Research Institute ‘Vall d’Hebron Institut de Recerca’.

**Guido Bertolini** is the Head of the Department of Medical Epidemiology at the Istituto di Ricerche Farmacologiche Mario Negri. He is coordinating the eCREAM (enabling Clinical Research in Emergency and Acute care Medicine through automated data extraction) project, which aims to develop new technical solutions to extract reliable clinical information from electronic patient files for scientific research.

**Maria Panagiotopoulou**, PhD in Biotechnology from the University of Technology of Compiègne, is a Senior Project Manager at ECRIN. Focused on applying FAIR principles in clinical research, she brings previous experience from her roles at the French National Centre of Scientific Research (CNRS) and the French Alternative Energies and Atomic Energy Commission (CEA).
